# Comparison of Multi- and Single-Site Robotic Myomectomy Using the Da Vinci^®^ SP Surgical System: A Propensity Score Matching Analysis

**DOI:** 10.3390/jcm11236905

**Published:** 2022-11-23

**Authors:** Jong Mi Kim, Yoon Hee Lee, Gun Oh Chong, Sae Rom Lee, Dae Gy Hong

**Affiliations:** 1 Department of Obstetrics and Gynecology, School of Medicine, Kyungpook National University, Daegu 41944, Republic of Korea; 2 Department of Obstetrics and Gynecology, Kyungpook National University Chilgok Hospital, Daegu 41404, Republic of Korea; 3 Clinical Omics Research Center, School of Medicine, Kyungpook National University, Daegu 41566, Republic of Korea

**Keywords:** robotic myomectomy, robotic multi-site myomectomy, robotic single-site myomectomy, da Vinci^®^ SP surgical system, propensity score matching

## Abstract

Objective: This study aimed to compare the surgical outcomes between robotic multi-site myomectomy (RMSM) and robotic single-site myomectomy (RSSM), using the da Vinci^®^ SP surgical system and perform propensity score matching analysis to ensure inter-group comparability. Methods: This retrospective study included 105 patients who underwent either three-incision RMSM or RSSM using the da Vinci^®^ SP surgical system. We retrospectively reviewed and compared surgical outcomes using 1:1 propensity score matching. Results: After 1:1 propensity score matching, there were no differences in the total operation time and estimated blood loss between the groups. The docking time (*p* < 0.0001) and duration of hospital stay (*p* = 0.0001) were significantly shorter in the RSSM group than in the RMSM group. Conclusions: The surgical outcomes of RSSM were comparable to those of RMSM. Moreover, compared to RMSM, RSSM using the da Vinci^®^ SP surgical system has shorter docking and morcellation times, and duration of hospital stay.

## 1. Introduction

Uterine myomas are one of the most common benign gynaecological tumours in women of reproductive age, with a prevalence of 25–40%. They cause menorrhagia, dysmenorrhoea, anaemia, pressure symptoms due to mass, and possible pregnancy-related complications [1,2]. Myomectomy is an alternative surgical method to hysterectomy that can be selected by women who want to preserve their fertility in the future [3].

Myomectomy using minimally invasive surgery techniques has several advantages, including faster recovery, shorter duration of hospital stay, and less post-operative pain, compared to laparotomy [4]. Single-port laparoscopic surgery was introduced to maximise the advantages of laparoscopic surgery. However, single-port laparoscopic myomectomy has several technical limitations, such as loss of triangulation, instrument crowding and clashing, and ergonomic difficulty.

Robotic-assisted surgery was developed to overcome the limitations of conventional laparoscopic surgery. In some complex cases and those involving atypical locations, such as deep intramural myomas, robot-assisted surgery can overcome the limitations of laparoscopy [5]. Since robotic single-site surgery (RSSS) using the da Vinci^®^ Si or Xi system (Intuitive Surgical Inc., Sunnyvale, CA, USA) was introduced in 2009, this approach has been applied to various gynaecological surgeries [6,7,8]. However, limited studies have evaluated single-site robotic myomectomy (RSSM). In a recent systematic review, Giovannopoulou et al. demonstrated the safety of RSSM and its equivalency to the multi-port technique [9]. However, the surgical performance of RSSM using the da Vinci^®^ Si or Xi system still has several limitations, such as difficulty in handling semi-rigid, single-site instruments, and restrictions in the range of motion [6,10]. In 2018, a fourth-generation model, the da Vinci^®^ SP surgical system, was developed. It contains four instrument drives that control the articulating camera and up to three robotic instruments that can be positioned simultaneously through a 25 mm single umbilical incision. Unlike previous models of da Vinci^®^ RSSS systems, the EndoWrist SP instruments have two joints. The wrist joint allows for 7° of freedom, and the elbow joint achieves intracorporeal triangulation in 6 mm, fully wristed, elbowed instruments. Therefore, the new da Vinci^®^ SP surgical system has sufficient articulation, appropriate power, and less crowding, and can overcome the limitations of RSSM using the da Vinci^®^ Si or Xi system. A recent pilot study reported that RSSM using the da Vinci^®^ SP surgical system, is a feasible surgical modality for women with symptomatic uterine myoma [11]. However, no study has compared multi-port robotic myomectomy and RSSM using the da Vinci^®^ SP surgical system.

Therefore, this study aimed to compare the surgical outcomes between robotic multi-site myomectomy (RMSM) and RSSM using the da Vinci^®^ SP surgical system, and perform propensity score matching analysis to ensure inter-group comparability.

## 2. Materials and Methods

### 2.1. Patient Selection and Clinicopathologic Characteristics

We retrospectively compared the data of 50 patients who underwent three-incision RMSM using the da Vinci^®^ Xi or Si surgical system (Intuitive Surgical, Inc.) and 55 patients who underwent RSSM using the da Vinci^®^ SP surgical system (Intuitive Surgical, Inc.). All surgeries were performed at Kyungpook National University Chilgok Hospital.

Retrospective data collection and analysis were approved by the Institutional Review Board of Kyungpook National University Chilgok Hospital (KNUCH2022-08-030). The requirement of informed consent was waived due to the retrospective nature of the study. All procedures were performed in accordance with the ethical standards of our institution and the Declaration of Helsinki 1964, and its later amendments.

The types and locations of uterine myomas on magnetic resonance images were classified according to the International Federation of Gynecologic Obstetrics (FIGO) classification [12] as follows: submucosal (FIGO type 0–2), intramural (FIGO type 3–4), subserosal (FIGO type 5–7), and others (FIGO type 8).

Estimated blood volume and total blood volume were calculated using the López-Picado formula and International Council for Standardization in Haematology formula [13].

### 2.2. Surgical Procedures

**Figure 1:**
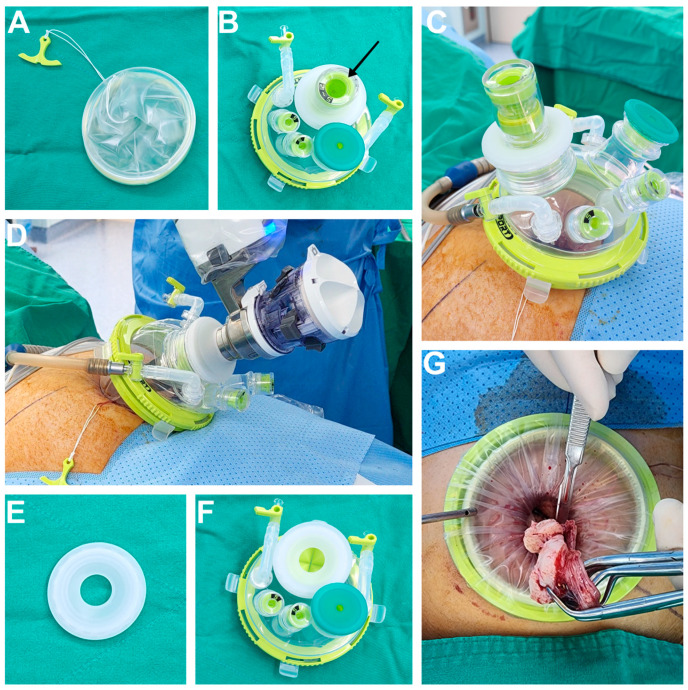
(**A**) The wound retractor part of the Uni-port. (**B**) The cap part of the Uni-port with a laparoscopic port cap (black arrow) and four additional ports. (**C**) The assembled Uni-port for laparoscopic surgery. The CO_2_ line is connected, and abdominal cavity is inflated. (**D**) da Vinci^®^ SP robot docking is completed. For robotic surgery, the port cap of the Uni-port is replaced, and the SP trocar is inserted. The CO_2_ line is connected, and the abdominal cavity is inflated. (**E**) The special port cap of the Uni-port for da Vinci^®^ SP robot trocar. (**F**) The cap part of the Uni-port for the da Vinci^®^ SP robot surgery system. The port cap indicated by the black arrow in Figure 1B has been replaced with the port cap for the da Vinci^®^ SP robot trocar. (**G**) After the resected myoma is placed in an endopouch, it is pulled by hand, by holding it with a tenaculum forceps or towel clip. The inlet part of the endopouch is covered and fixed to the retractor part of the Uni-port. Subsequently, manual morcellation using scalpel is performed.

The surgical procedure for RSSM was as follows. Under general anaesthesia, the patient was positioned to the lithotomy and Trendelenburg position. A uterine manipulator was inserted into the uterine cavity, via the transvaginal route in sexually active patients. A vertical umbilical skin incision of approximately 25–30 mm in length was made, extending to the peritoneum. We used a Uni-port (DalimSurgNet, Seoul, Korea) specially designed for the da Vinci^®^ SP surgical system. The Uni-port consists of two parts—a flexible wound retractor (Figure 1A) and cap with four ports (one 15 mm port, one 28 mm port for the da Vinci^®^ SP trocar, and two additional 5 mm ports) (Figure 1B). No additional skin incision was made for the secondary trocar. Instead, the Uni-port’s 15 mm and 5 mm trocars were used for assistance, such as during suction and irrigation, and the introduction of needles or gauze. The wound retractor was inserted into the opening of the incision using Army-Navy retractors and the port cap was fixed to the wound retractor (Figure 1C). The abdomen was then insufflated with CO_2_ gas, and a laparoscope was inserted to identify the location of myoma and pelvic adhesions. Since there was no robotic instrument available for strong traction in the SP system, we used a hybrid technique with laparoscopy for myoma enucleation, before the docking of the robotic surgical system. First, a diluted vasopressin 0.25 U/mL concentrate solution was injected into the myoma capsule. Then, uterine incision and resection of the myoma were performed using a Harmonic scalpel (Ethicon, Somerville, NJ, USA). Traction was performed using a laparoscopic myoma screw. After enucleation of the myoma, the da Vinci^®^ SP surgical system robot was docked with a single arm using four instrument devices on the right side of the patient (Figure 1D). There was a special port cap for the 28 mm port in the Uni-port (Figure 1E), making it possible to switch between laparoscopic surgery and robotic surgery by changing the port cap, without removing the main cap from the wound retractor (Figure 1F). The cut edge of the myometrium and uterine serosa was sutured in at least two to four layers using continuous and/or interrupted 1-0 to 3-0 absorbable suture materials. The enucleated myomas were contained in the endopouch and removed through the umbilical trocar site using in-bag manual morcellation with a scalpel (Figure 1G).

The surgical procedure for RMSM was similar to that for RSSM. However, for RMSM, a vertical umbilical skin incision of approximately 20–25 mm in length was made, shorter than RSSM, but sufficient for RMSM. The RMSM required two additional 8 mm lateral trocars placed 8–10 cm lateral to the umbilicus, for the two robotic arms. The da Vinci^®^ Xi or Si was docked with three arms at the foot of the bed. The other techniques used for myoma enucleation and morcellation were the same as those used for RSSM.

### 2.3. Statistical Analysis

Differences between subsets were calculated using Student’s *t*-test or the Mann-Whitney test, and differences between proportions were evaluated using the chi-square test. MedCalc^®^ statistical software (version 20.110, MedCalc Software Ltd., Ostend, Belgium) and IBM SPSS^®^ statistical software (version 26.0, IBM Corp. Released 2019, Armonk, NY, USA) were used for statistical analyses. A *p*-value of <0.05 was considered statistically significant. Marginal significance was defined as a *p*-value of 0.05–0.10.

Further, 1:1 propensity score matching was performed for 50 patients in the RMSM group and 55 patients in the RSSM group using IBM SPSS. Patient preoperative characteristics, age, body mass index (BMI), weight of myomas, number of myomas, size of dominant myoma, FIGO type of dominant myoma, and surgical history, were used for propensity score matching. In total, 26 pairs were matched and compared.

## 3. Results

### 3.1. Clinicopathological Characteristics

The clinicopathological characteristics of patients and myoma characteristics for all study participants are provided in Table 1. There was no significant difference between the RMSM and RSSM groups in terms of mean age. However, the mean BMI (21.39 ± 2.29 kg/m^2^ vs. 23.76 ± 4.57 kg/m^2^, *p* = 0.0010) and proportion of patients with a history of surgery (6 [12%] vs. 18 [32.7%], *p* = 0.0119), were significantly higher in the RSSM group than in the RMSM group. The characteristics of uterine myoma, including weight, FIGO classification, and total number, were similar between the groups (Table 1).

### 3.2. Surgical Outcomes

There were no differences in estimated blood loss and number of patients who had intraoperative or post-operative complications, between the RMSM and RSSM groups. However, the RSSM group showed better surgical outcomes in terms of the total operation time (135.67 ± 41.00 min vs. 172.82 ± 77.96 min, *p* = 0.0023), docking time (2.98 ± 1.83 min vs. 7.08 ± 1.66 min, *p* < 0.0001), morcellation time (4.68 ± 6.54 min vs. 10.66 ± 10.83 min, *p* = 0.0046), and duration of hospital stay (3.33 ± 0.86 days vs. 4.42 ± 1.37 days, *p* < 0.0001), than the RMSM group.

### 3.3. Matched Sample

The patient and myoma characteristics of the two groups were similar after 1:1 propensity score matching (Table 2). There were no significant differences in the total operation time, or estimated blood loss between the groups. Console time (53.96 ± 27.97 min vs. 67.85 ± 28.79 min, *p* = 0.0942) and manual morcellation time (3.07 ± 4.27 min vs. 6.81 ± 6.22 min, *p* = 0.0525) were shorter in the RSSM group than in the RMSM group, with marginal significance. Moreover, docking time (3.04 ± 2.03 min vs. 6.73 ± 1.69 min, *p* < 0.0001) and duration of hospital stay (3.12 ± 0.77 days vs. 4.54 ± 1.48 days, *p* = 0.0001) were significantly shorter in the RSSM group than in the RMSM group (Table 2). Intra-operative and post-operative complications were similar between the groups, before and after 1:1 propensity score matching (Table 1 and Table 2).

## 4. Discussion

To the best of our knowledge, this is the first study to compare surgical outcomes between RSSM using the da Vinci^®^ SP surgical system and RMSM. After 1:1 propensity score matching, the surgical outcomes of the RSSM and RMSM groups were comparable. Furthermore, the duration of hospital stay and docking time were significantly shorter in the RSSM group, than in the RMSM group.

Several studies have compared surgical outcomes between RMSM and RSSM. In a multi-centre retrospective comparative study performed by Moawad et al., after adjustment for multiple covariates with regression models, RSSM and RMSM had comparable estimated blood loss (83.3 mL vs. 109.2 mL, *p* = 0.34), operative time (162.4 min vs. 162.4 min, *p* = 0.99), overnight admission (odds ratio [OR] = 1.54, *p* = 0.44) and post-operative complications (OR = 1.3, *p* = 0.78) [14]. Further, Lee et al. reported similar results, showing that the total operative time, estimated blood loss, difference in haemoglobin levels, transfusion rate, and post-operative fever did not differ between RSSM and RMSM [15]. However, Ahn et al. concluded that the total operative time was significantly shorter in the RSSM group than in the RMSM group (150.9 ± 57.1 min vs. 170 ± 74.5 min, *p* = 0.0296) [16]. Moreover, the visual analogue scale pain score was significantly lower in the RSSM group than in the RMSM group (2.4 ± 0.8 vs. 2.7 ± 0.8, *p* = 0.0149) [16]. Furthermore, Won et al. reported that the RSSM group had a shorter operative time (148.3 ± 44.8 min vs. 162.3 ± 47.4 min, *p* = 0.011), lesser decrease in haemoglobin levels (1.8 ± 0.9 g/dL vs. 2.3 ± 1.0 g/dL, *p* < 0.001), and shorter duration of hospital stay (5.4 ± 0.7 days vs. 5.8 ± 0.7 days, *p* < 0.001) than the RMSM group [17]. In our study, the total operative time, docking time, console time, time to manual morcellation, and duration of hospital stay were significantly shorter in the RSSM group than in the RMSM group before 1:1 propensity score matching. Moreover, the difference in haemoglobin levels was significantly lower in the RSSM group than in the RMSM group (*p* = 0.0023). After 1:1 propensity score matching, the docking time, time to morcellation, and duration of hospital stay were significantly shorter in the RSSM group than in the RMSM group.

In our study, we used a hybrid technique for RSSM—myoma enucleation using laparoscopy, and suture using a robot. Sufficient traction strength is essential to completely enucleate a myoma. However, RSSSs do not have appropriate instruments for traction of the myoma, such as a tenaculum. In conventional laparoscopic myomectomy, sufficient strength is provided using a tenaculum or myoma screw. To overcome the problem of traction in RSSM, a hybrid technique may be useful. This hybrid technique may maximise the advantages of laparoscopic and robotic surgical techniques during RSSM.

The da Vinci^®^ SP surgical system was docked on the right side of the patient, and the da Vinci^®^ SP camera and various EndoWrist SP instruments were inserted through the holes of the entry guide kit into a single incision site. Docking was performed simply and quickly using the da Vinci^®^ SP surgical system. In our study, docking time was significantly shorter in the RSSM group than in the RMSM group (3.04 ± 2.03 min vs. 6.73 ± 1.69 min. *p* < 0.0001). In cases of extraction of multiple myomas using the hybrid technique, numerous switches between laparoscopic and robotic surgical techniques may be needed, and a fast docking time may be helpful in reducing the total operation time.

Myoma removal is another time-consuming step. The United States Food and Drug Administration has issued a safety communication urging doctors not to use laparoscopic power morcellation for hysterectomies or removal of uterine myomas, due to concerns that the technique may disseminate uterine sarcomas beyond the uterus [18]. In the case of RMSM, power morcellation is usually performed using a 12 mm trocar that is positioned in the upper abdomen. Despite several studies introducing the surgical technique of in-bag power morcellation to prevent spillage of tissue fragments, the procedure of in-bag power morcellation is somewhat technically difficult and time-consuming. Therefore, we previously introduced robotic myomectomy without power morcellation, using a single-port assisted three-incision technique with in-bag manual morcellation [19]. Since a single-port incision site provides a larger opening for specimen removal, it is convenient to use a bag that allows morcellations to be performed more safely without concern about the spread of malignant particles [20]. Moreover, it is possible to remove myomas rapidly with a scalpel without using power morcellation, which enables a shorter operation time while preventing potential injuries caused by the morcellator [21,22]. In our study, RMSM was performed using a single-port assisted three-incision technique with in-bag manual morcellation. Because the da Vinci^®^ SP surgical system had a somewhat bulky entry guide kit in the single incision site, the length of the single site of the RSSM (2.5–3 cm) was slightly longer than that of the RMSM (2–2.5 cm). This may explain the shorter time to manual morcellation of RSSM, compared to that of RMSM, in our study (3.07 ± 4.27 min vs. 6.81 ± 6.22, *p* = 0.0525).

A recent systematic review and meta-analysis showed that pain score was significantly lower in the single-port laparoscopic myomectomy group, than in the conventional laparoscopic myomectomy group [23]. In addition, Ahn et al. demonstrated that the post-operative pain score was significantly lower in RSSM than in RMSM (2.7 ± 0.8 vs. 2.4 ± 0.8, *p* = 0.0149) [17]. In our study, the duration of hospital stay in the RSSM group was significantly shorter than in the RMSM group (3.12 ± 0.77 days vs. 4.54 ± 1.48, *p* = 0.0001). Although we did not evaluate post-operative pain score, fast recovery due to lower post-operative pain might have affected the shorter duration of hospital stay in the RSSM group.

This study has several limitations. First, it was a retrospective study that included a small study population. Second, it was a single-centre study; the generalization is partly limited. Nonetheless, this study had several strengths. To our knowledge, this is the first study to compare the surgical outcomes of RMSM and RSSM using the da Vinci^®^ SP surgical system. Additionally, 1:1 propensity score matching was performed to reduce inter-group bias.

## 5. Conclusions

After 1:1 propensity score matching, the surgical outcomes of RSSM and RMSM groups were comparable. Moreover, RSSM using the da Vinci^®^ SP surgical system was associated with shorter docking and morcellation times, and duration of hospital stay.

## Figures and Tables

**Table 1:** Clinicopathologic characteristics and surgical outcomes of robot myomectomy before 1:1 propensity score matching.

Variables	Multi-Port (*n* = 50)	Single-Port (*n* = 55)	*p* Value
Mean age (years)	36.26 ± 7.15	38.13 ± 6.20	0.155
Body mass index (kg/m^2^)	21.39 ± 2.29	23.76 ± 4.57	0.001
Previous abdominal surgery history (*n*, %)	6 (12%)	18 (32.7%)	0.012
Total operation time (min)	172.82 ± 77.96	135.67 ± 41.00	0.002
Docking time (min)	7.08 ± 1.66	2.98 ± 1.83	<0.001
Console time (min)	89.52 ± 51.00	60.51 ± 29.97	0.001
Laparoscope time (min)	69.04 ± 48.24	71.13 ± 30.18	0.949
Time to manual morcellation (min)	10.66 ± 10.83	4.68 ± 6.54	0.005
Estimated blood loss (mL)	457.81 ± 310.95	380.43 ± 235.09	0.157
Haemoglobin change (g/dL)	1.61 ± 0.81	1.56 ± 0.81	0.002
Duration of hospital stay (days)	4.42 ± 1.37	3.33 ± 0.86	<0.001
Intraoperative complications (*n*, %)			1.000
Rectal serosa injury	1 (2.0)	0 (0.0)	
Small bowel adhesiolysis and suture	0 (0.0)	1 (1.8)	
Post-operative complications (*n*, %)			0.667
Post-operative fever	3 (6.0)	0 (0.0)	
Wound disruption	0 (0.0)	1 (1.8)	
Incisional hernia	0 (0.0)	1 (1.8)	
Type of main myoma (*n*, %)			0.113
Submucosal (FIGO type 0–2)	0 (0.0)	4 (7.3)	
Intramural (FIGO type 3–4)	29 (58.0)	25 (45.5)	
Subserosal (FIGO type 5–7)	20 (40.0)	22 (40.0)	
Others (FIGO type 8)	1 (2.0)	4 (7.3)	
Total number of uterine myomas (*n*)	3.90 ± 3.72	2.76 ± 2.16	0.062
Total weight of uterine myomas (g)	251.66 ± 213.57	197.00 ± 192.71	0.141

FIGO = International Federation of Gynaecology and obstetrics.

**Table 2:** Clinicopathologic characteristics and surgical outcomes of robot myomectomy after 1:1 propensity score matching.

Variables	Multi-Port (*n* = 26)	Single-Port (*n* = 26)	*p* Value
Mean age (years)	37.96 ± 8.04	37.15 ± 5.21	0.669
Body mass index (kg/m^2^)	21.66 ± 2.14	21.64 ± 2.23	0.984
Previous abdominal surgery history (*n*, %)	5 (19.23)	8 (30.77)	0.341
Total operation time (min)	143.50 ± 50.22	129.92 ± 44.05	0.305
Docking time (min)	6.73 ± 1.69	3.04 ± 2.03	<0.001
Console time (min)	67.85 ± 28.79	53.96 ± 27.97	0.094
Laparoscope time (min)	62.12 ± 31.29	68.43 ± 33.17	0.496
Time to manual morcellation (min)	6.81 ± 6.22	3.07 ± 4.27	0.053
Estimated blood loss (mL)	442.55 ± 292.36	383.59 ± 222.64	0.417
Haemoglobin change (g/dL)	1.84 ± 0.74	1.59 ± 0.86	0.274
Duration of hospital stay (days)	4.54 ± 1.48	3.12 ± 0.77	0.001
Intraoperative complications (*n*)	0	0	1.000
Post-operative complications (*n*, %)			
Post-operative fever	3 (11.5)	0 (0.0)	0.235
Type of main myoma (*n*, %)			0.212
Submucosal (FIGO type 0–2)	0 (0.0)	2 (7.7)	
Intramural (FIGO type 3–4)	18 (69.2)	14 (53.8)	
Subserosal (FIGO type 5–7)	8 (30.8)	8 (30.8)	
Others (FIGO type 8)	0 (0.0)	2 (7.7)	
Total number of uterine myomas (*n*)	2.92 ± 2.10	2.31 ± 1.59	0.178
Total weight of uterine myomas (g)	166.10 ± 168.70	197.35 ± 220.34	0.568

FIGO = International Federation of Gynaecology and obstetrics.

## Data Availability

The data that support the findings of this study are available from the corresponding author upon reasonable request.
